# Thirty Years and Beyond: Long-term Outcomes After Liver Transplantation at a Single Center: Progress, Challenges, and Future Implications

**DOI:** 10.1097/TXD.0000000000002013

**Published:** 2026-09-22

**Authors:** Nora Nevermann, Anna Riddermann, Ulrike Bode, Philipp Felgendreff, Markus Quante, Nicolas Richter, Linda Feldbrügge, Heiner Wedemeyer, Richard Taubert, Dieter Haffner, Eva-Doreen Pfister, Ulrich Baumann, Katrin Splith, Moritz Schmelzle, Burckhardt Ringe

**Affiliations:** 1 Department of General, Visceral and Transplant Surgery, Hannover Medical School, Hannover, Germany.; 2 Clinical Scientist Program PRACTIS, Carl-Neuberg-Straße 1, Hannover, Germany; 3 Department of Gastroenterology, Hepatology, and Endocrinology, Hannover Medical School, Hannover, Germany.; 4 Department of Pediatric Kidney, Liver, Metabolic and Neurological Diseases, Hannover Medical School, Hannover, Germany.; 5 Division of Pediatric Gastroenterology and Hepatology, Department of Pediatric Kidney, Liver, Metabolic, and Neurological Diseases, Hannover Medical School, Hannover, Germany.

## Abstract

**Background.:**

The liver transplantation (LT) program was established at Hannover Medical School in 1972. The aim of the study was to assess graft and patient survival, organ function, and active life throughout ≥3 decades after LT, with a special focus on recipients surviving ≥30 y after transplantation.

**Methods.:**

Clinical data from all consecutive patients undergoing LT between 1972 and 1994 were collected in a prospective design, and outcome data were complemented retrospectively.

**Results.:**

Nine hundred LTs performed on 756 patients were included in the study. Survival increased with growing experience during the study period. One hundred thirty-eight patients survived ≥30 y, of whom 35 (25%) received >1 graft. Chronic kidney disease was observed in 96 recipients (70%), necessitating additional kidney transplants in 16 patients (12%). De novo malignancy was diagnosed in 31 patients (23%; 4% after pediatric and 33% after adult LT, *P* < 0.001) during the long-term follow-up. The leading cause of death beyond a 30-y survival was cardiovascular. Twenty-seven patients (38%) had a child after LT; 33 pediatric transplant recipients (89%) engaged in professional employment posttransplant.

**Conclusions.:**

This is the first study offering detailed insight into LT survival beyond 30 y. The data show that 30-y survival after LT is reachable in relevant numbers, with stable graft function and the opportunity to live an active, self-directed life.

## Graphical abstract

**Figure:**
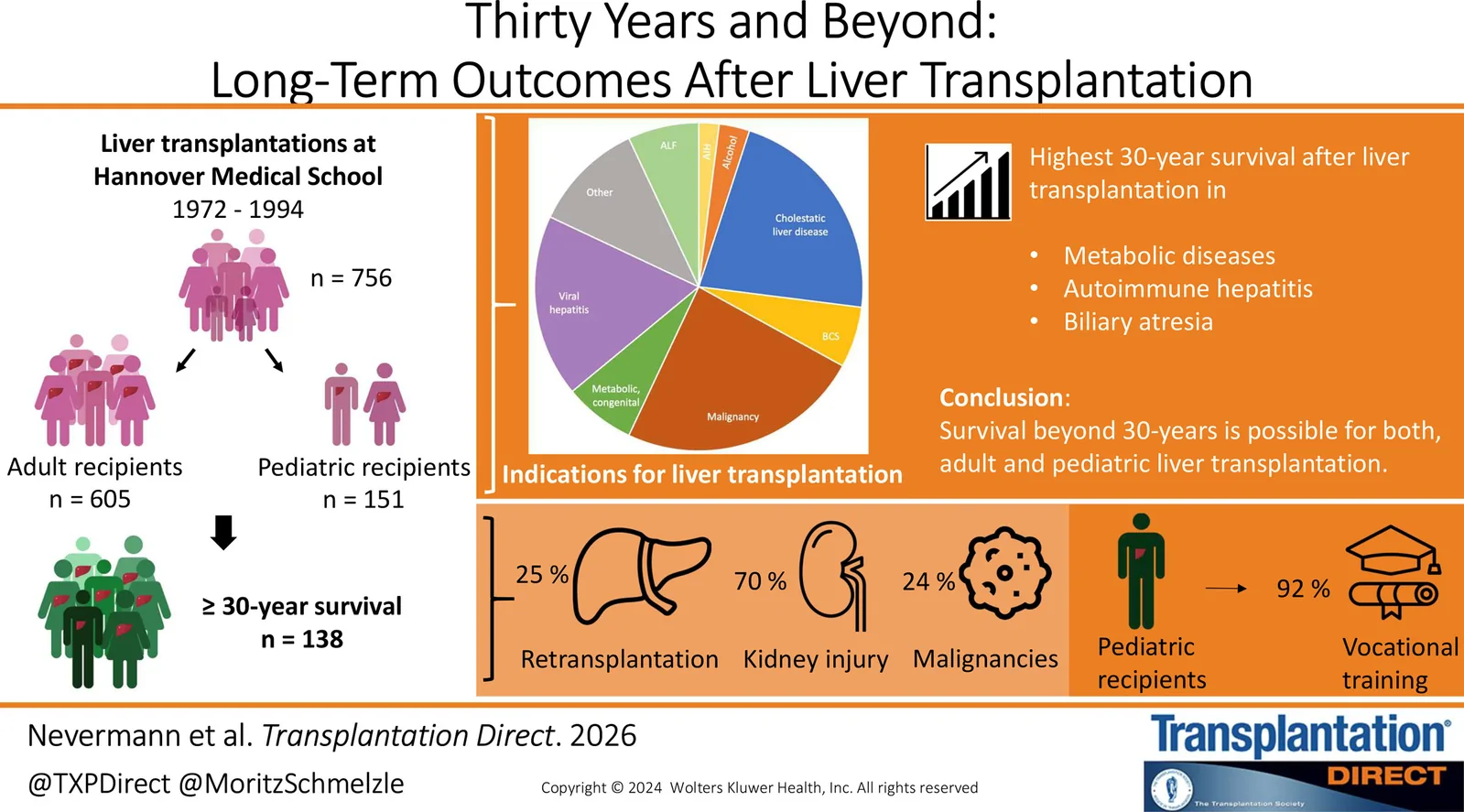

## INTRODUCTION

With the first liver transplantation (LT) performed in 1972, Hannover Medical School (MHH) aligned with 3 institutions, Denver/Pittsburgh (United States), Cambridge (United Kingdom), and Groningen (the Netherlands), among the pioneering centers for LT. The 4 centers presented their initial transplant experience during the first consensus development conference on LT held in Bethesda (United SA) in 1983.^1^

Based on the long-standing transplant program of MHH, the opportunity now arises to obtain detailed insights into transplant survival exceeding 30 y. This unique cohort therefore provides the opportunity not only to analyze patient and graft survival but also to characterize the long-term medical and functional consequences of transplantation across 3 decades.

Although LT has evolved into the standard treatment for end-stage liver disease, current knowledge regarding patients surviving ≥30 y after LT remains limited. Most published studies focus predominantly on short- and mid-term outcomes, whereas the clinical course of survivors beyond 3 decades after transplantation is still insufficiently characterized.^2-6^ Consequently, important questions remain unanswered regarding long-term graft function, causes of late mortality, retransplantation, and the functional and social reintegration of transplant recipients after decades of variant immunosuppressive therapy.

We therefore conducted a study reviewing all LTs performed until 1994, with the potential for a follow-up of at least 30 y. The primary focus of this analysis was the cohort of recipients who survived ≥30 y after LT. This unique cohort provides the first comprehensive insight into graft and patient survival, organ function, late mortality, retransplantation, and active life throughout 3 decades. By specifically focusing on 30-y survivors, this study aims to address an important gap in the current literature and better define the clinical reality of life ≥30 y after LT.

## MATERIALS AND METHODS

### Study Design

Clinical data of all consecutive patients undergoing LT at the Department of General, Visceral and Transplant Surgery, Hannover Medical School (MHH), until January 1994, were collected in a prospective design, and outcome data were complemented retrospectively.

The study was approved by the MHH Institutional Review Board (vote number 11436_BO_K_2024), waiving the need for written informed consent from the patients. All data were collected, stored, and processed in accordance with local data protection regulations. The study was conducted in accordance with the ethical standards of the Declaration of Helsinki, as revised in 2024.

### Data Collection on the Overall LT Cohort

Data on the overall LT cohort were systematically collected and they included the following: recipient age and gender, donor characteristics (age, sex, size, weight, cold ischemia time, cause of death), transplantation indications, surgical techniques, data on retransplantation where applicable, and data on follow-up and survival. For adult transplant recipients, additional clinical variables included pretransplant diabetes mellitus, cardiovascular comorbidities, history of portal vein thrombosis, smoking status at transplantation, and renal function during the initial posttransplant hospitalization. Recipient age of 18 y or older was defined as an adult transplantation. Partial LT (pLT) or split LT were defined as variant transplantations. Lost to follow-up was defined as an interval of >5 y after the last recorded patient contact.

Nine main LT indication categories were defined on the basis of frequency: alcohol-related cirrhosis, autoimmune hepatitis (AIH), acute cryptogenic or drug-induced liver failure, cholestatic liver disease, Budd-Chiari syndrome, malignancy, metabolic/congenital diseases, viral hepatitis, and other; malignancies were further subdivided by entity, and causes within cholestatic liver disease were explicitly delineated.

### Data Collection and Outcome Parameters for the 30-y Survivors

**FIGURE 1.**
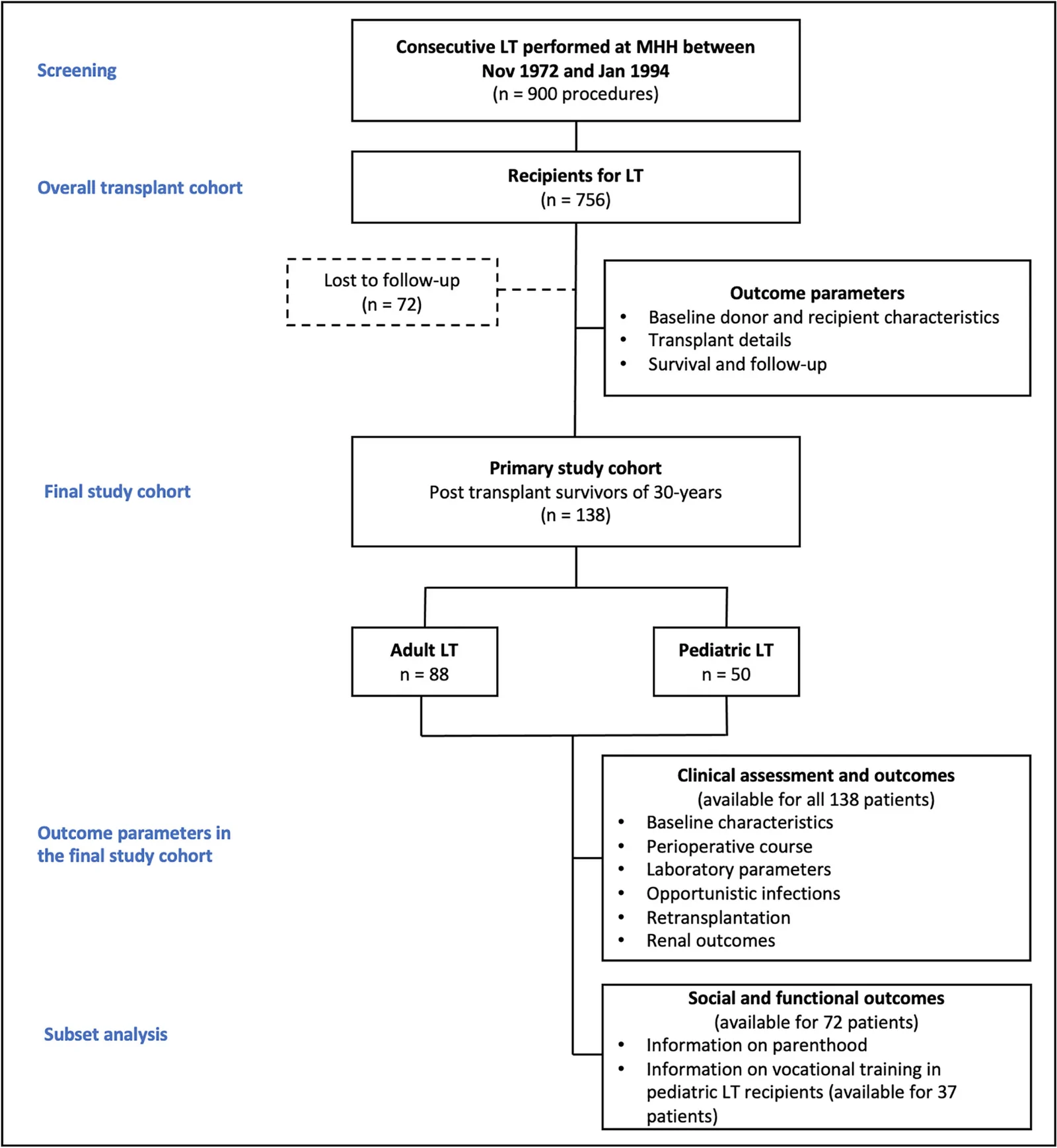
Flowchart of the study design. LT, liver transplantation; MHH, Hannover Medical School.

Particular emphasis was placed on recipients who survived for at least 30 y after LT, who constituted the primary study cohort (Figure 1). For these patients, baseline characterization as described earlier was complemented by data on medical history before LT, physical status at LT, intraoperative and postoperative results, and medical events throughout follow-up. Postoperative complications were recorded and graded according to the Clavien-Dindo classification, with severe complications defined as Clavien-Dindo grade >IIIa.^7^ Kidney injury was defined according to the Staging Criteria for Acute Kidney Injury (2012) as KDIGO G2 or higher. Laboratory values for kidney and liver function at 10, 20, and 30 y after LT were collected. The occurrence of opportunistic infections was documented during the posttransplant follow-up period. This included viral infections such as cytomegalovirus (CMV), Epstein-Barr virus, varicella zoster virus, and herpes simplex virus; bacterial infections, including Yersiniosis; invasive fungal infections such as oral and esophageal candidiasis; and parasitic infections, notably toxoplasmosis. Information on parenthood was collected from 30-y survivors. Information on vocational training and professional occupation was obtained for patients after pediatric LT.

### Surgical Techniques of LT Throughout the Study Period

**FIGURE 2.**
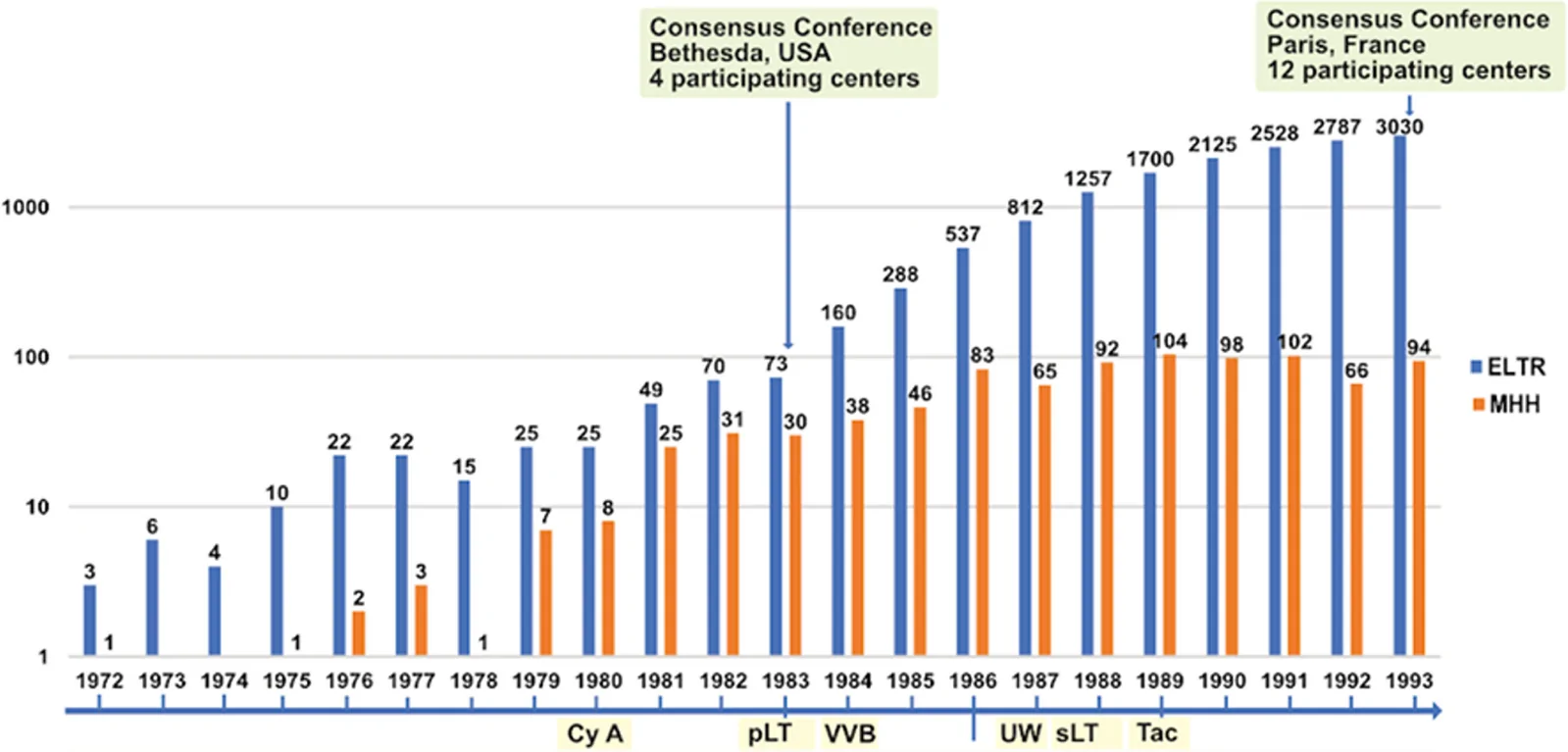
Evolution of LT in Europe and Hannover between 1972 and 1993, indicating major events and the clinical introduction of new immunosuppressive drugs. Cy A, cyclosporine A; ELTR, European Liver Transplant Registry; LT, liver transplantation; pLT, partial LT; sLT, split LT; Tac, tacrolimus; UW, University of Wisconsin perfusion solution; VVB, veno-venous bypass.

Beginning in August 1984, veno-venous bypass was adopted as the standard technique. For whole-organ LT, replacement of the inferior vena cava (IVC) was performed as a standard procedure. The piggyback technique conserving the IVC was applied only twice during the study period in whole-organ LTs. Until 1983, pLT with replacement of the IVC was performed in pediatric LT to overcome the discrepancy between the size of an adult donor liver and the pediatric recipient. pLT (segments II + III) with end-to-side anastomosis of the left hepatic vein was performed for the first time in 1983.^1^ Figure 2 shows the transplant volume at MHH compared with worldwide transplantation numbers during the study period and highlights the milestones in surgical and perioperative technique development.

### Immunosuppression and Follow-up After LT

The initial immunosuppressive regimen comprised high-dose prednisolone (1000 mg bolus with tapering), azathioprine (Imurek® 100 mg), and antilymphocyte serum. By 1980, the regimen had evolved to a triple therapy with prednisolone, cyclosporine A (used off-label before official approval in 1982), and antithymocyte globulin. In 1986, lymphocyte-specific antibodies were introduced, with BT563 (anti-interleukin-2 receptor monoclonal antibody) implemented in 1990 and subsequently adopted as routine induction therapy. The advent of tacrolimus (FK506) in October 1990 marked a pivotal advancement in immunosuppression.

### Statistical Analysis

Categorical variables were presented using frequencies and proportions, whereas continuous variables were reported as median and range. The Wilcoxon rank-sum test was used to analyze continuous variables, whereas the Pearson chi-square test was used for categorical variables. Multivariable Cox proportional hazards regression analyses were performed a priori to identify independent predictors of posttransplant survival. The model included recipient characteristics at the time of transplantation (age, gender, cardiovascular comorbidities, diabetes mellitus, portal vein thrombosis, and smoking status), recipient renal function during the initial posttransplant hospitalization, as well as donor characteristics (age, sex, weight, and height) as covariates. In addition, univariable Cox proportional hazards regression analyses were performed for each of these covariates to evaluate their unadjusted association with posttransplant survival. Kaplan-Meier analysis was conducted and compared with the log-rank test using GraphPad Prism Version 9.3.1 (GraphPad Software, San Diego, CA). A *P* value of <0.05 was considered statistically significant. Data analysis was performed using IBM SPSS Statistics Version 25.0 (IBM Corp, Armonk, NY).

## RESULTS

Between November 1972 and January 1994, 900 LTs were performed on 756 patients at MHH. Of those, 72 patients were lost to follow-up (Figure 1). Twenty percent of all included LTs were pediatric LTs (n = 152). Baseline characteristics and technical parameters for the overall LT cohort are summarized in Table 1.

**TABLE 1.** Baseline characteristics for overall LT population

	All (N = 756)	Pediatric (n = 152)	Adult (n = 604)
No. of grafts			
1	613 (81%)	111 (73%)	502 (83%)
2	124 (16%)	37 (24%)	87 (14%)
3	18 (2%)	4 (2.6%)	14 (2%)
4	1	–	1
LT with veno-venous bypass	388 (51%)	15 (10%)	373 (62%)
Recipient characteristics			
Age, y	38 y (0–74)	5 y (0–17)	42 y (18–74)
Sex (male/female)	377 (50%)/379 (50%)	81 (53%)/71 (47%)	296 (49%)/308 (51%)
Donor characteristics			
Age	23 y (0–63)	9 y (0–52)	25 y (1–63)
Missing	10 (1%)	6 (4%)	4 (1%)
Sex (male/female)	380 (63%)/224 (37%)	77 (65%)/42 (35%)	303 (62%)/182 (38%)
Missing	152 (20%)	33 (22%)	119 (20%)
Weight, kg	70 (7–120)	30 (7–90)	70 (20–120)
Missing	160 (21%)	36 (24%)	124 (21%)
Height, cm	172 (46–204)	140 (46–195)	175 (100–204)
Missing	179 (24%)	44 (29%)	135 (22%)
Cause of death			
TBI	346 (58%)	74 (61%)	272 (56%)
SAH	96 (16%)	11 (9%)	85 (18%)
ICH	107 (18%)	19 (16%)	88 (18%)
Hypoxia	22 (4%)	9 (8%)	13 (3%)
Stroke	11 (2%)	2 (2%)	9 (2%)
Other	20 (3%)	5 (4%)	15 (3%)
missing	154 (21%)	32 (21%)	122 (20%)

Values are stated as median (range) or number (%). Missing values are stated in numbers (%). Donor characteristics refer to the first liver graft of all 756 patients.

ICH, intracranial hemorrhage; LT, liver transplantation; SAH, subarachnoid hemorrhage; TBI, traumatic brain injury.

**FIGURE 3.**
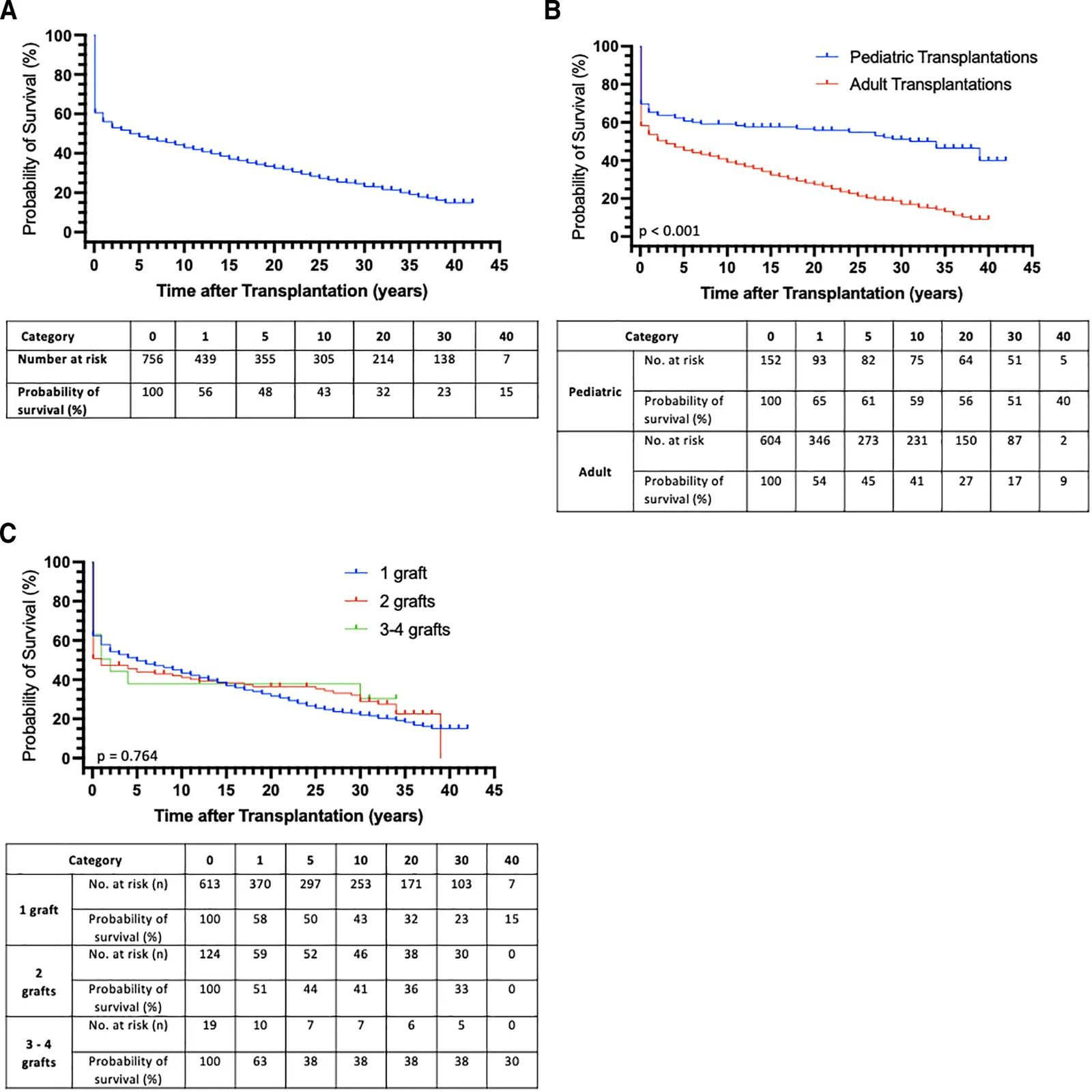
Patient survival after LT between November 1972 and January 1994. *P* value: log-rank comparison of survival curves. A, Overall patient survival after LT. B, Patient survival in adult and pediatric recipients. C, Recipient survival compared by the number of grafts per patient.

**FIGURE 4.**
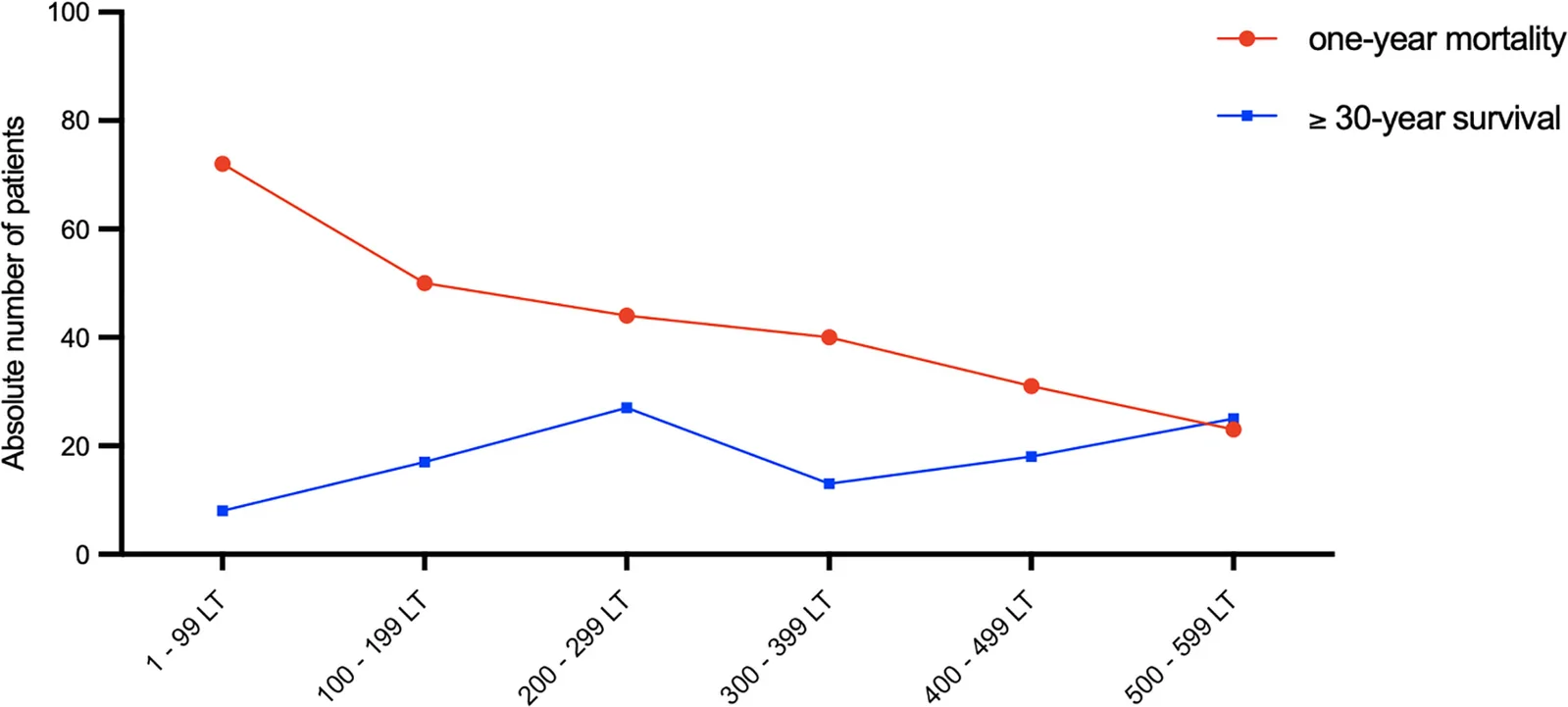
Absolute numbers of 1-y mortality and ≥30-y survival after LT at the center, shown across increasing cumulative transplant volumes (0–99 LT, 100–199 LT, etc) since program initiation. LT, liver transplantation.

Overall patient survival rates at 1, 10, 20, and 30 y were 58%, 40%, 28%, and 18%, respectively, as determined by Kaplan-Meier analysis (Figure 3A). The evolution of 1-y mortality and 30-y survival rates by cumulative experience is illustrated in Figure 4.

In patients who survived the first year posttransplantation, median survival was 31 y (1–42 y) for pediatric recipients and 16 y (1–40 y) for adult recipients. Kaplan-Meier survival curves for these groups are shown in Figure 3B. Median survival for patients with survival beyond 1 y receiving 1, 2, or 3 to 4 grafts was 17 (1–42 y), 30 (1–39 y), and 21 y (1–34 y; log-rank *P* = 0.764). Kaplan-Meier survival curves for these groups and according to the number of liver grafts are shown in Figure 3C.

**FIGURE 5.**
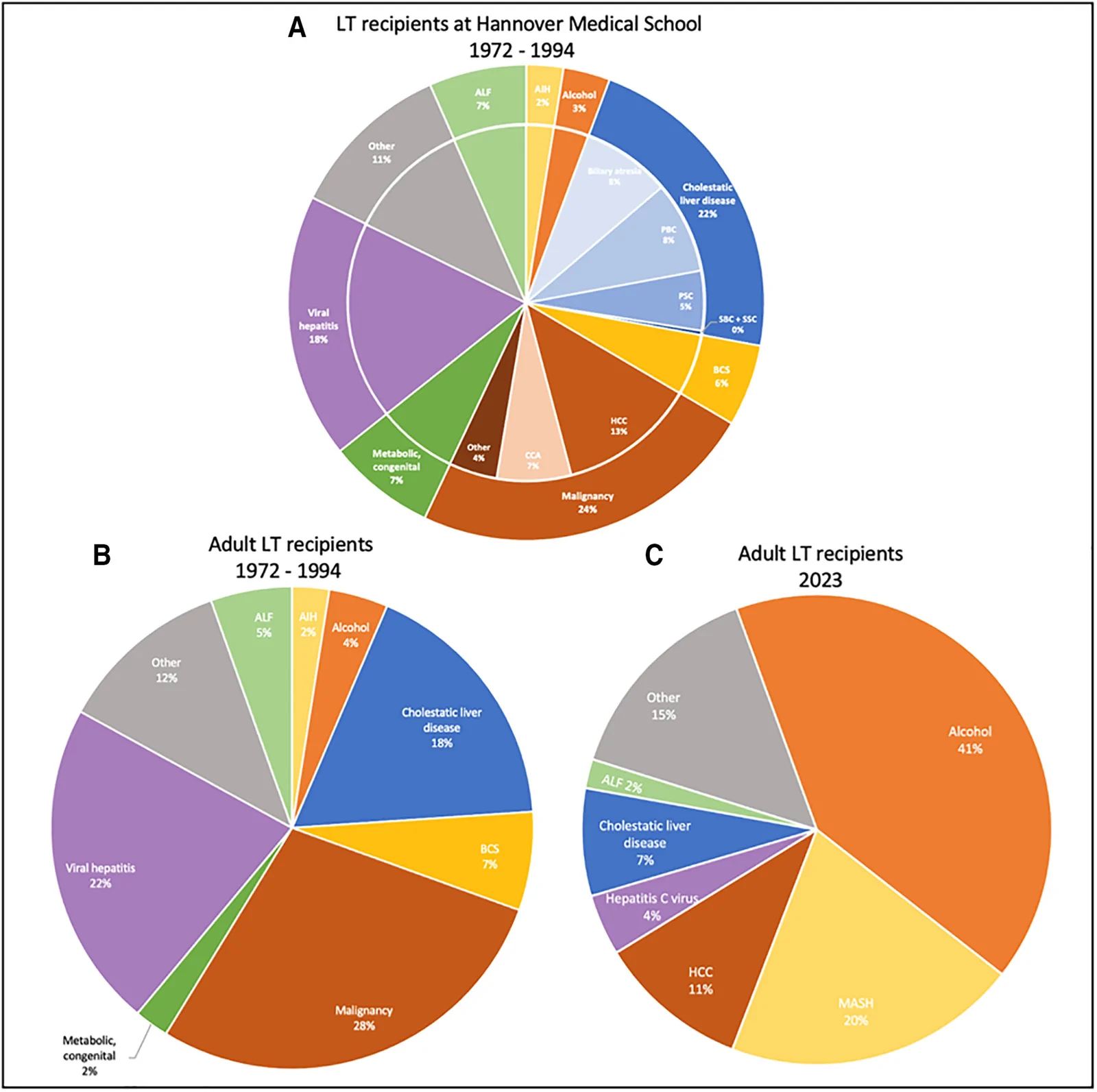
Indications for LT during the study period compared with the current practice. A, All LT recipients, 1972–1994. B, Adult LT recipients, 1972–1994. C, Adult LT recipients, 2023. (14) AIH, autoimmune hepatitis; ALF, acute liver failure; BCS, Budd-Chiari syndrome; CCA, cholangiocarcinoma; HCC, hepatocellular carcinoma; LT, liver transplantation; PBC, primary biliary cholangitis; PSC, primary sclerosing cholangitis; SBC, secondary sclerosing cholangitis; SSC, secondary sclerosing cholangitis.

**FIGURE 6.**
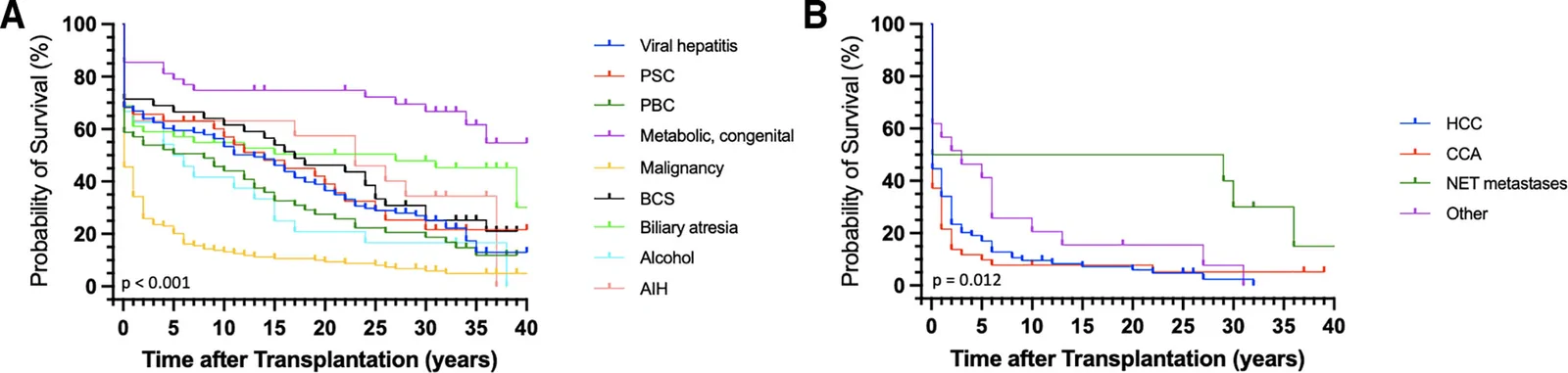
Patient survival after LT (November 1972–January 1994). *P* value from log-rank test comparing survival curves. A, By indication for liver transplantation. B, For malignant entities. LT, liver transplantation.

The most frequent indications for LT were malignant diseases, followed by cholestatic liver diseases and viral hepatitis (Figure 5). The highest 30-y survival rates were observed in patients transplanted for metabolic diseases (46%), followed by AIH (37%), biliary atresia (31%), and Budd-Chiari syndrome (26%; Figure 6A). Among malignant diseases, neuroendocrine tumor (NET) metastases yielded the highest 30-y survival at 13% (Figure 6B). For viral hepatitis, the survival of chronic hepatitis C and B was compared, which showed no difference in Kaplan-Meier analysis (log-rank p = 0.921; **Figure S1, SDC,** https://links.lww.com/TXD/A911).

Univariable Cox proportional hazards regression analyses for adult transplant recipients are presented in **Table S1** (**SDC,** https://links.lww.com/TXD/A912). In the univariable analyses, preexisting cardiovascular comorbidities, diabetes mellitus, higher early posttransplant serum creatinine levels, older recipient age, and male recipient gender were associated with significantly poorer posttransplant survival. However, in the multivariable Cox regression analysis, preexisting cardiovascular comorbidities (hazard ratio [HR], 1.33; 95% confidence interval [CI], 0.89-1.97; *P* = 0.161), diabetes (HR, 1.33; 95% CI, 0.85-2.07; *P *= 0.209), portal vein thrombosis (HR, 0.96; 95% CI, 0.56-1.66; *P* = 0.892), smoking before transplantation (HR, 1.16; 95% CI, 0.77-1.74; *P* = 0.484), recipient gender (HR, 1.10; 95% CI, 0.86-1.41; *P* = 0.442), donor age (HR, 1.00; 95% CI, 0.99-1.01; *P *= 0.987), donor gender (HR, 1.16; 95% CI, 0.86-1.55; *P* = 0.331), donor weight (HR, 1.00; 95% CI, 0.98-1.01; *P* = 0.687), and donor height (HR, 1.01; 95% CI, 0.99-1.02; *P* = 0.503) were not identified as significant predictors of posttransplant survival. In contrast, recipient age at transplantation (HR, 1.02; 95% CI, 1.01-1.04; *P* < 0.001) and renal function during the initial posttransplant hospitalization (HR, 1.001; 95% CI, 1.000-1.002; *P *= 0.003) emerged as independent predictors of posttransplant survival.

Among the 756 transplant recipients, 138 (18%) achieved a posttransplant survival of 30 y. These cases define the study cohort.

### Perioperative Outcome in 30-y Survivors

Pretransplantation characteristics of 30-y survivors are shown in Table 2. A median postoperative liver enzyme peak for alanine aminotransferase was documented at 590 U/L (100–3157 U/L) and for aspartate aminotransferase at 426 U/L (72–4444 U/L) for the 30-y survivors. Of the 138 patients, 1 underwent split LT (0.7%) and 13 underwent pLT (9.4%). The median length of hospital stay was 44 days (24–251 d). Severe postoperative complications occurred in 55 cases (40%). Relaparotomy was necessary in 46 cases (33%), and retransplantation within the same hospital stay was performed in 15 patients (11%).

**TABLE 2.** Pretransplantation characteristics of 30-y survivors

	All (N = 138)	Pediatric (n = 50)	Adult (n = 88)
Recipient characteristics			
Age, y	25 y (0–61)	6 y (0–17)	36 y (18–61)
Sex (male/female)	61 (45%)/76 (55%)	27 (55%)/23 (45%)	34 (39%)/54 (61%)
Diabetes	3 (4%)	0 (0%)	3 (7%)
Missing	61 (44%)	18 (36%)	43 (49%)
MELD score			
<15	21 (15%)	8 (16%)	13 (15%)
15–27	37 (27%)	15 (30%)	22 (25%)
>27	19 (14%)	9 (18%)	10 (11%)
Missing	61 (44%)	18 (36%)	43 (49%)
Recipient clinical history			
Previous surgery	59 (43%)	23 (46%)	36 (41%)
Disease-related	47 (80%)	21 (91%)	26 (72%)
Exploratory laparotomy	12 (26%)	7 (33%)	4 (15%)
Exploratory laparoscopy	6 (13%)	1 (5%)	5 (19%)
Partial liver resection	3 (6%)	0 (0%)	3 (12%)
Portocaval shunt	4 (9%)	0 (0%)	4 (15%)
Kasai procedure/BDA	15 (32%)	10 (48%)	5 (19%)
Other	7 (15%)	2 (10%)	5 (19%)
Non-disease related	12 (20%)	2 (9%)	10 (28%)
Esophageal variceal bleeding prior LT	36 (46%)	6 (18%)	30 (67%)
Missing	60 (44%)	17 (34%)	43 (49%)
Encephalopathic episode prior LT	16 (32%)	3 (19%)	13 (31%)
Missing	80 (58%)	34 (68%)	46 (52%)
Cause of liver disease			
Viral hepatitis	28 (20%)	0 (0%)	28 (32%)
Metabolic disorder	25 (18%)	19 (38%)	6 (7%)
Biliary atresia	19 (14%)	19 (38%)	0 (0%)
BCS	11 (8%)	1 (2%)	10 (11%)
PBC	11 (8%)	0 (0%)	11 (13%)
Malignant tumor	8 (6%)	2 (4%)	6 (7%)
PSC	7 (5%)	0 (0%)	7 (8%)
AIH	6 (4%)	2 (4%)	4 (5%)
Alcohol	3 (2%)	0 (0%)	3 (3%)
Other	20 (14%)	7 (14%)	13 (15%)
Sub/acute liver failure	17 (12%)	6 (12%)	12 (14%)
Donor characteristics			
Age	19 y (1–62)	8 y (1–44)	22 y (7–62)
Missing	1 (0.7%)	1 (2%)	0 (0%)
Sex (male/female)	75 (65%)/41 (35%)	24 (62%)/15 (38%)	51 (66%)/26 (34%)
Missing	22 (16%)	11 (22%)	11 (13%)
Weight, kg	65 (10–100)	25.5 (10–90)	70 (20–100)
Missing	23 (17%)	12 (24%)	11 (13%)
Height, cm	170 (46–195)	135 (46–185)	175 (110–195)
Missing	29 (21%)	17 (35%)	12 (14%)

Values are stated as median (range) or number (%). Missing values are stated in numbers (%). Donor characteristics refer to the first liver graft of all 138 patients.

AIH, autoimmune hepatitis; BCS, Budd-Chiari syndrome; BDA, biliodigestive anastomosis; LT, liver transplantation; MELD, Model for End-Stage Liver Disease; PBC, primary biliary cirrhosis; PSC, primary sclerosing cholangitis .

### Long-term Outcome in 30-y Survivors

**FIGURE 7.**
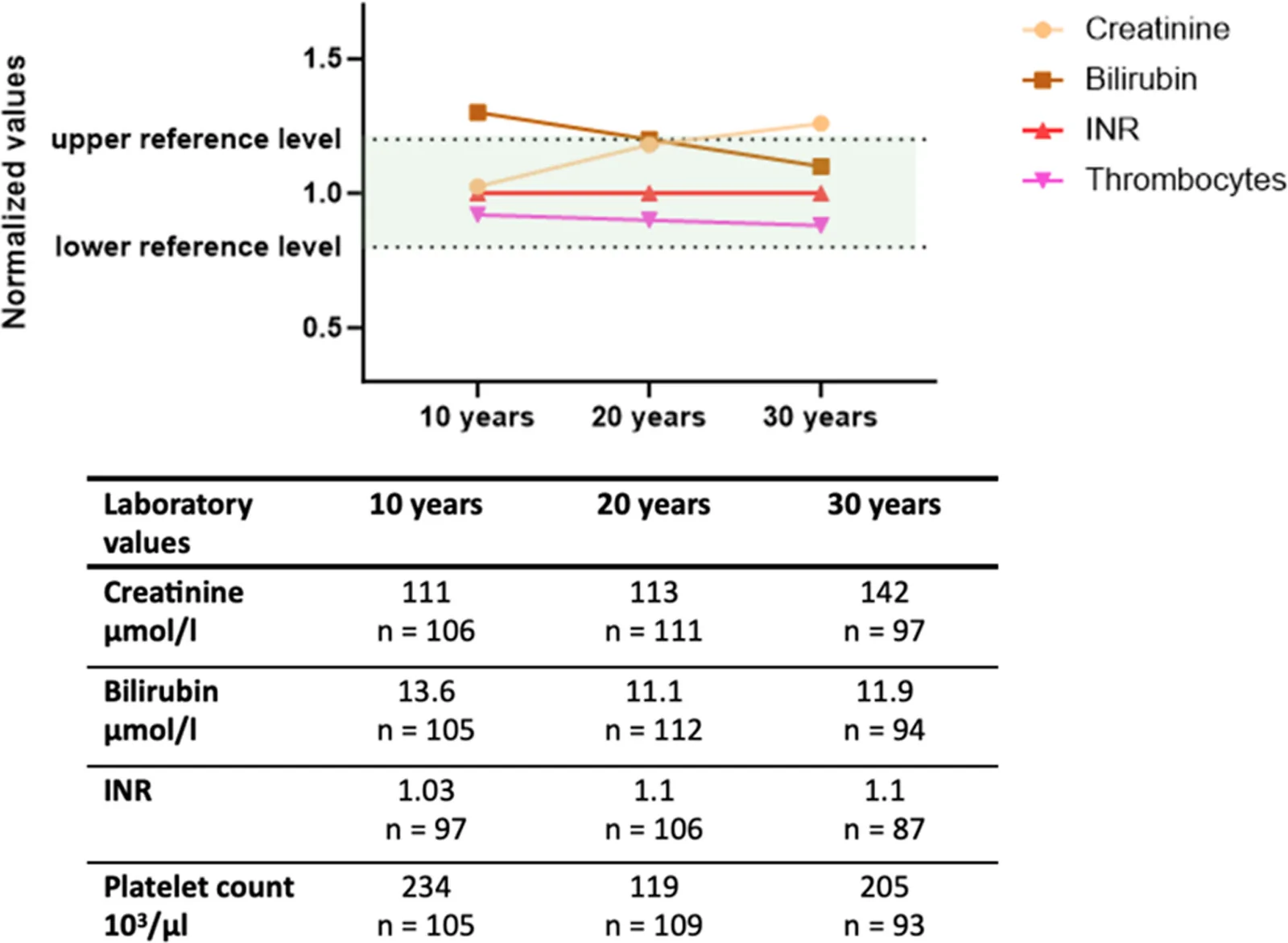
Laboratory values 10, 20, and 30-y after LT. Values were calculated as means and expressed as normalized values (0.8 defined as lower reference level and 1.2 upper reference level). INR, international normalized ratio; LT, liver transplantation.

During the long-term follow-up, 35 (25%) retransplantations were required. The most common indication for retransplantation was chronic transplant rejection, followed by recurrent cholangitis. Opportunistic infections occurred in 22 patients (16%), representing 10 pediatric (20%) and 12 adult transplant recipients (14%; *P* = 0.326; Table 3). CMV reactivation was reported for 9 patients (4 pediatric LT, 5 adult LT). Three cases of CMV hepatitis were reported at 2, 6, and 10 y after LT, and 1 case of CMV colitis 2 y after LT. One case of Epstein-Barr virus reactivation was observed (adult LT). Impaired renal function over time after LT was observed in 96 liver recipients (70%), necessitating ≥1 kidney transplantation in 16 patients (12%). Laboratory parameters, including creatinine levels, bilirubin levels, INR, and platelet count, were evaluated at 10, 20, and 30 y after LT. Among these, only serum creatinine levels showed a progressive increase over time (Figure 7). Malignant diseases were diagnosed in 33 patients (24%) during the long-term follow-up, including 31 (21%) de novo and 2 (1%) recurrent malignancies (Table 3). The most common de novo malignancy was skin cancer (16 cases; 52%), followed by breast cancer (7 cases; 23%) and renal cancer (6 cases; 19%). Among the 16 patients with skin tumors, 9 (56%) had cutaneous squamous cell carcinoma, 6 (38%) had basal cell carcinoma, and 2 (13%) had melanoma. One patient was diagnosed with 2 distinct skin tumors. De novo malignancies occurred significantly more frequently in adults compared with children (29 [33%] versus 2 [4%], *P* < 0.001). Of the 138 patients, 28 had died at the time of analysis, with 5 deaths attributed to graft-related causes (Table 3).

**TABLE 3.** Posttransplant complications in 30-y survivors

	All (N = 138)	Pediatric (n = 50)	Adult (n = 88)
Graft-related outcomes			
Retransplantation	35/138 (25%)	13/50 (26%)	22/88 (25%)
Hepatitis reinfection	6/28 (25%)	0/0 (0%)	6/28 (21%)
Posttransplant complications			
Infection	22/138 (16%)	10/50 (20%)	12/88 (14%)
Kidney injury^*a*^	96/138 (70%)	34/50 (68%)	62/88 (71%)
Kidney transplantation	16/138 (12%)	4/50 (8%)	12/88 (14%)
Malignancy			
Any malignancy	33/138 (24%)	3/50 (6%)	30/88 (34%)
Recurrent tumor	2/138 (1%)	1/50 (2%)	1/88 (1%)
De novo tumor^*b*^	31/138 (23%)	2/50 (4%)	29/88 (33%)
Long-term mortality			
Mortality after 30 y	28/138 (20%)	3/50 (6%)	25/88 (28%)
Cardiovascular	7/28 (25%)	1/3 (33%)	6/25 (24%)
Graft-related mortality	5/28 (18%)	2/3 (66%)	3/25 (12%)
Renal	4/28 (14%)	0/3 (0%)	4/25 (16%)
De novo malignancy	3/28 (11%)	0/3 (0%)	3/25 (12%)

Values are stated as number (%). *P* = Significance according to Pearson and chi-square test.

^*a*^Kidney Injury defined as KDIGO (2012) ≥G2, and the KDIGO 2024 update.

^*b*^Skin cancer (16/31), breast cancer (7/31), and renal cancer (6/31).

KDIGO, Kidney Disease Improving Global Outcome.

### Long-term Social and Functional Outcomes After LT in 30-y Survivors

Information on social and functional outcomes, including parenthood and vocational training after transplantation, was available for 72 patients (52%). Of those, 27 patients (38%) had children posttransplantation, including 18 female and 9 male recipients. The highest number of children after transplantation per patient was 6 for male and 5 for female recipients. No case of infertility was reported.

Among the 37 pediatric recipients for whom information on vocational training was accessible, 34 (92%) completed vocational training, and 9 patients (24%) completed a university degree. A total of 33 patients (89%) were engaged in professional employment.

## DISCUSSION

In this study, we present data on long-term LT outcomes with follow-up periods of ≥30 y. The study complements existing data on outcomes up to 20 y after transplantation.^2-6,8-11(p20)^ Ten- and 20-y survival rates of 61% and 41%–43% have been reported.^8,12^ Only recently have data on patient survival beyond 30 y after LT become available, emphasizing the rarity of such long-term analyses and highlighting the importance of cohorts with extended follow-up.^13^

By nature of the large follow-up interval, our study is based on a transplant cohort up to 1994. The development of surgical techniques and standardized care are outlined previously. The evolution of the 1-y mortality (Figure 4) attests to the surgical and medical progress during our study period. Despite the “historical” framing that inevitably comes along with the long follow-up interval, our cohort provides valuable insights into LT survival beyond 30 y that have not yet been reported.

These advancements likely reflect the cumulative effect of overcoming the initial learning curve of LT, alongside improved recipient selection and optimized transplant indications, all contributing to enhanced patient outcomes.^12,14^ This finding is consistent with the observations reported by Bowing et al in pediatric LT.^11^ Based on our calculations, at least one-third of currently transplanted patients can be expected to survive for ≥30 y—possibly more as continuous improvements continue. Donor-related factors such as age and weight did not predict long-term survival in multivariable analysis. This finding suggests a potentially greater influence of recipient characteristics and posttransplant management on outcomes.

The most frequent indications for adult LT were malignant diseases, viral hepatitis, followed by cholestatic liver diseases (Figure 5). These have since been largely supplanted by alcoholic liver disease, metabolic dysfunction-associated steatohepatitis, and hepatocellular carcinoma as dominant indications in contemporary practice (Figure 5).^15^ Although hepatocellular carcinoma has become the dominant LT indication supported by specialized MELD exception calculations to optimize posttransplant survival, our survival curves demonstrate comparable outcomes for patients with cholangiocarcinoma.^15^ The development of protocol-based selection criteria, such as the Mayo protocol for perihilar CCA, has significantly improved patient selection and survival in this historically challenging indication.^16^ Emerging criteria for intrahepatic CCA are currently under refinement.^17^

As already reported by data on 20-y survival, a superior outcome for pediatric compared with adult LT is further confirmed by our study.^2,5^ In our historical cohort, a 30-y survival probability of 51 for pediatric versus 17% for adult LT is shown (Figure 3B). This difference has been attributed to the lower burden of comorbidities in infants and to differing indications for LT.^5^ These age groups differ substantially in terms of underlying disease spectrum and regenerative capacity.^18^ As emphasized by Jain et al., pediatric recipients represent a distinct long-term survivor population with fewer age-related comorbidities and lower competing mortality risks than adult recipients.^19^ They were therefore analyzed separately in the present study.

Interestingly, our data show no trend for inferior survival among patients necessitating (multiple) retransplantation (*P* = 0.764; Figure 3C). Five of the 19 cases that required 3 or 4 grafts survived ≥30 y (probability of survival: 38%; Figure 3C). In the context of increasing organ shortages, this finding suggests a justified deployment of multiple grafts for 1 recipient despite medical and technical challenges. These results diverge from previous reports in the literature, which have described significantly poorer overall survival after retransplantation.^5,20^ This discrepancy may, at least in part, be attributable to the limited sample size in our subgroup analysis and should therefore be interpreted with caution.

Median donor age for the overall LT cohort (n = 756) was 23 y (0–63 y). The Organ Procurement and Transplantation Network/Scientific Registry of Transplant Recipients 2023 annual report on LT reported that only 27.3% of deceased liver donors were older than 55 y, underscoring the relevance and comparability of our cohort to contemporary practice.^15^ Although the proportion of elderly donors is increasing, contemporary machine perfusion techniques can substantially improve organ function, making meaningful correlation with our cohort feasible.

Zooming in on the study cohort of patients with a survival of 30 y, several conclusions can be drawn from our data. When evaluating organ function, stable laboratory values for the synthetic and excretory functions of the liver are seen during the decades, as shown in Figure 7, in accordance with Schoening and Duffy et al.^2,9^ In contrast, renal function decreases steadily. Independent of the age at LT, a high percentage of patients develop kidney injury, with around 10% of all patients requiring kidney transplantation during the course of decades. Similar percentages of patients requiring kidney transplantation 25 y after LT have been described before.^21^ These numbers have to be put in the context of the lacking evidence on optimal dosage of immunosuppression at the time of transplantation. Cyclosporine A was used as standard immunosuppression until the introduction of tacrolimus and is considered a major risk factor for kidney injury. Its nephrotoxic effect was uncovered in the 1990s.^22^ Besides cyclosporine A, other important risk factors for chronic kidney injury after LT are hypertension, hepatitis C infection, diabetes mellitus, and postoperative acute renal failure.^23^ Posttransplant renal dysfunction has consistently emerged as one of the major determinants of very long-term outcome. In the recently published 30-y analysis by Jiménez-Romero et al,^13^ chronic renal impairment was identified as an independent predictor of late mortality, underlining the importance of nephroprotective immunosuppressive strategies throughout long-term follow-up.

In contrast to the homogeneous distribution of kidney injury, malignancy—and especially multiple malignancies—is observed at a higher rate for adult LT compared with pediatric LT after 30 y. Although 3 of 50 (6%) patients after pediatric LT developed a malignant disease, 30 of 88 patients (34%) receiving LT in adulthood reported a malignant disease. After skin cancer, breast cancer was the second leading de novo malignancy in patients after LT, which is consistent with previous findings.^24^ The incidence of non-melanoma skin cancer in our cohort was substantially higher, reaching 11%, compared with rates reported after 20 y of follow-up in previous studies.^19^ These findings align with the observations of Dopazo et al,^25^ who reported preserved long-term graft function among 20-y survivors despite a steadily increasing burden of extrahepatic comorbidities, particularly malignancies, emphasizing that long-term morbidity is also increasingly driven by non-graft-related complications. Our data extend these observations by demonstrating that this burden continues to rise beyond 3 decades after transplantation. Our data thus suggest a relevant role for common carcinogenic factors enhanced by decades-long immunosuppressive medication. Carcinogenesis induced by a synergistic effect of immunosuppression and lifestyle factors can hereby be presumed, resulting in a significantly increased cancer incidence observed in adult transplant recipients.^26^

The leading cause of death after the 30-y threshold was found to be cardiovascular. Twenty-eight deaths were recorded beyond 30-y survival after LT. Seven of those were due to cardiovascular incidents and were therefore the largest group, resembling statistics of the general Western population.^27^ In frequency, cardiovascular deaths were followed by graft-related deaths, deaths due to renal complications, and malignancies. As already demonstrated by Adam et al,^8^ technical complications and rejections play minor roles in mortality decades after LT, whereas malignancies and general causes of death become increasingly important. Further analysis of this finding beyond description will require larger case numbers.

Long-term management of LT recipients should focus on the prevention, early detection, and treatment of chronic comorbidities that emerge with prolonged graft survival. As survival extends across decades, patients are less frequently limited by graft failure and more by the cumulative burden of cardiovascular disease, renal dysfunction, metabolic complications, malignancies, and long-term effects of immunosuppression. Structured follow-up care aimed at maintaining overall health rather than solely preserving graft function is indispensable.

Finally, our data show that beyond a vast numerical survival, the transplant recipients were able to lead active, self-directed lives, including vocational training, professional employment, and parenthood.

As already evoked, several relevant limitations have to be considered when reading the results of our study. Most importantly, the historical context of the surgical and perioperative state of knowledge has to be considered. Direct transferability of our results to a recent transplant cohort does not appear to be appropriate. Beyond knowledge and medical inventions, the donor characteristics also differ largely from today’s reality. Furthermore, our data reflect a single high-volume center and its early experiences. As a next step, confirmation of our results in a multi-institutional design is required. Due to the retrospective nature of this analysis, the long observation period, and the fact that some patients received further treatment at external institutions, missing data and follow-up—particularly regarding laboratory parameters—were inevitable.

## CONCLUSIONS

Despite the mentioned limitations, the large cohort from the early MHH transplant program allows to show that survival beyond 30 y after LT is possible for both adult and pediatric LT. Hereby, our study offers detailed insights into 30-y survival. During 3 decades after transplantation, survival is reachable in relevant numbers and with stable graft function and the opportunity to live an active and self-directed life. Although kidney injury represents a relevant and frequent complication with equal distribution among pediatric and adult LT, a presumably synergistic effect of immunosuppression and common carcinogenic factors results in a statistically higher cancer rate in adult LT recipients. A stringent surveillance schedule appears to be highly warranted.

## ACKNOWLEDGMENTS

The authors want to acknowledge the founder of the transplant program at Hannover Medical School, the late Rudolf Pichlmayr, as well as other earlier contributors Christoph E. Broelsch, Peter Neuhaus, and Martin Burdelski.

We further acknowledge the support by PRACTIS Clinician Scientist Program, funded by Hannover Medical School and DFG (DFG 3696/3).

## Supplementary Material


