# Supplementary material for: Thirty Years and Beyond: Long-term Outcomes After Liver Transplantation at a Single Center: Progress, Challenges, and Future Implications
Source: Transplant Direct. 2026 Sep 22;12(10):e2013. doi: 10.1097/TXD.0000000000002013 (PMC13600577; doi:10.1097/TXD.0000000000002013)
Supplement: Supplementary file 1 [file txd-12-e2013-s001.pdf]

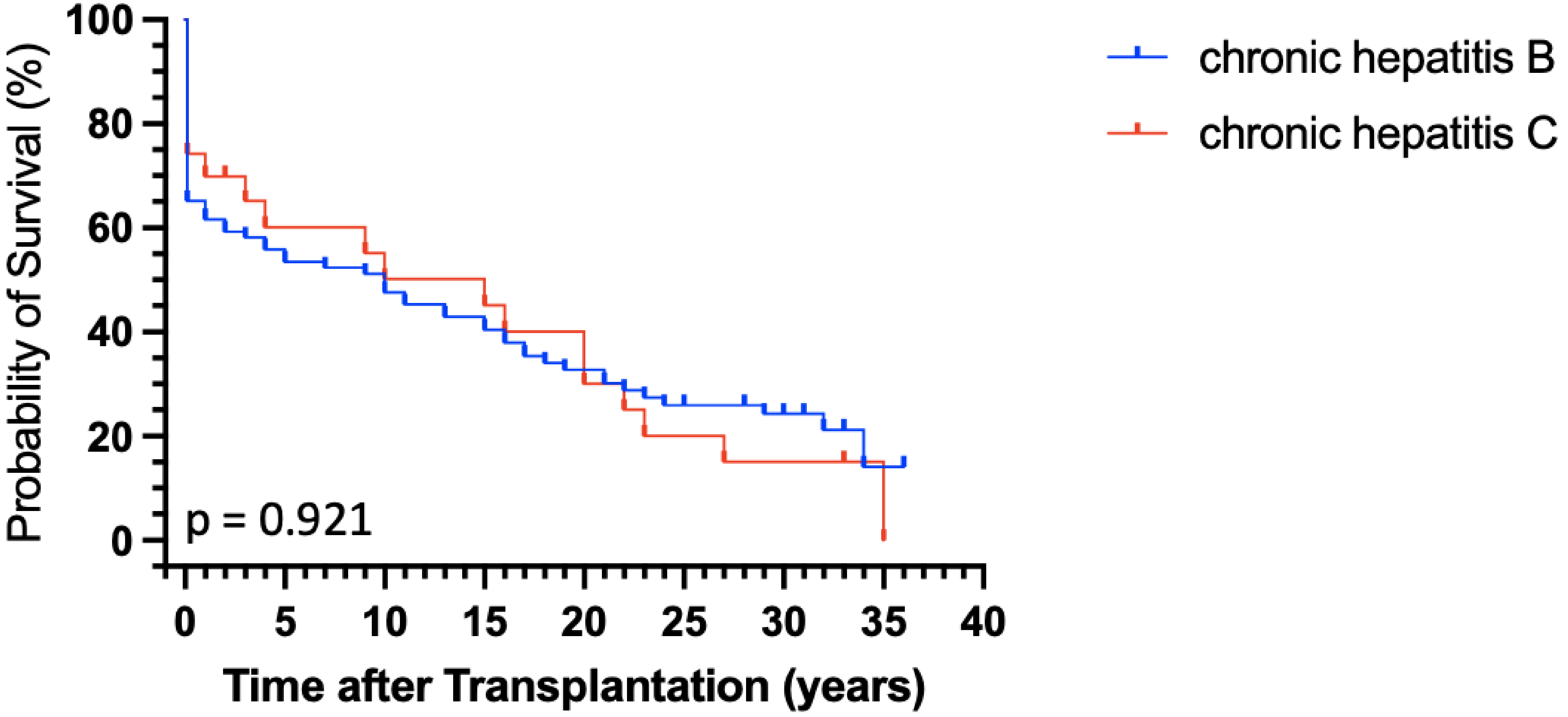

Probability of Survival (%)
100
80
60
40
20
0
p = 0.921
0
5
10
15
20
25
30
35
40
Time after Transplantation (years)
chronic hepatitis B
chronic hepatitis C
