# Supplementary material for: Thirty Years and Beyond: Long-term Outcomes After Liver Transplantation at a Single Center: Progress, Challenges, and Future Implications
Source: Transplant Direct. 2026 Sep 22;12(10):e2013. doi: 10.1097/TXD.0000000000002013 (PMC13600577; doi:10.1097/TXD.0000000000002013)
Supplement: Supplementary file 2 [file txd-12-e2013-s002.pdf]

Table S1: Recipient and donor characteristics included in the Cox proportional hazards regression analyses and results of univariable Cox proportional hazards regression analyses for post-transplant survival.

Values are presented as median (range) or number (%). Missing values are reported as numbers (%).

	**Adult (n = 604)**	**Univariable HR (95 % CI)**	**p**
**Recipient characteristics**			
Age	36 years (18 - 61)	1.02 (1.01 - 1.03)	<0.001
Gender (male vs. female)	296 (49 %) / 308 (51 %)	1.23 (1.03 - 1.47)	0.023
**Recipient clinical history**			
Cardiovascular comorbidities	47 / 530 (9 %)	1.71 (1.26 - 2.32)	<0.001
Diabetes	36 / 529 (7 %)	1.55 (1.10 - 2.19)	0.012
Portal vein thrombosis	30 / 529 (6 %)	0.83 (0.54 - 1.27)	0.379
Smoking	48 / 528 (9 %)	1.04 (0.75 - 1.44)	0.823
Early post-tranplant creatinin level (µmol/l)	175 (25 - 849)	1.00 (1.00 - 1.00)	<0.001
missing	88 (15 %)		
**Donor characteristics**			
Age	25 years (1 - 63)	1.04 (1.00 - 1.01)	0.352
missing	4 (1 %)		
Gender (male vs. female)	303 (62 %) / 182 (38 %)	1.11 (0.90 - 1.36)	0.335
Weight (kg)	70 (20 - 120)	1.00 (0.99 - 1.01)	0.757
missing	124 (21 %)		
Height (cm)	175 (100 - 204)	1.00 (0.99 - 1.01)	0.978
missing	135 (22 %)		
